# Aft1 Nuclear Localization and Transcriptional Response to Iron Starvation Rely upon TORC2/Ypk1 Signaling and Sphingolipid Biosynthesis

**DOI:** 10.3390/ijms24032438

**Published:** 2023-01-26

**Authors:** Sandra Montellà-Manuel, Nuria Pujol-Carrion, Maria Angeles de la Torre-Ruiz

**Affiliations:** Cell Signalling in Yeast Unit, Department of Basic Medical Sciences, Institut de Recerca Biomèdica de Lleida (IRBLleida), University of Lleida, 25198 Lleida, Spain

**Keywords:** TORC2, Ypk1, iron deprivation, Aft1, iron homeostasis, sphingolipids, LCBs, CK2, survival, inositol phosphorylceramide (IPC)

## Abstract

Iron scarcity provokes a cellular response consisting of the strong expression of high-affinity systems to optimize iron uptake and mobilization. Aft1 is a primary transcription factor involved in iron homeostasis and controls the expression of high-affinity iron uptake genes in *Saccharomyces cerevisiae*. Aft1 responds to iron deprivation by translocating from the cytoplasm to the nucleus. Here, we demonstrate that the AGC kinase Ypk1, as well as its upstream regulator TOR Complex 2 (TORC2), are required for proper Aft1 nuclear localization following iron deprivation. We exclude a role for TOR Complex 1 (TORC1) and its downstream effector Sch9, suggesting this response is specific for the TORC2 arm of the TOR pathway. Remarkably, we demonstrate that Aft1 nuclear localization and a robust transcriptional response to iron starvation also require biosynthesis of sphingolipids, including complex sphingolipids such as inositol phosphorylceramide (IPC) and upstream precursors, e.g., long-chain bases (LCBs) and ceramides. Furthermore, we observe the deficiency of Aft1 nuclear localization and impaired transcriptional response in the absence of iron when TORC2-Ypk1 is impaired is partially suppressed by exogenous addition of the LCB dihydrosphingosine (DHS). This latter result is consistent with prior studies linking sphingolipid biosynthesis to TORC2-Ypk1 signaling. Taken together, these results reveal a novel role for sphingolipids, controlled by TORC2-Ypk1, for proper localization and activity of Aft1 in response to iron scarcity.

## 1. Introduction

Iron is indispensable for life in every organism and plays an essential role in cellular processes such as respiration, DNA synthesis and repair, and diverse metabolic reactions as an essential cofactor [1,2]. Iron levels must also be tightly regulated as elevated levels have toxic effects, including contributing to the production of reactive oxygen species (ROS) [3,4]. However, insufficient iron levels also cause detrimental effects within cells [5]. Indeed, iron deficiency is the most prevalent nutritional disorder in the world with consequences on human health that are well established [6]. Iron trafficking and metabolism require tight regulation, and studies in budding yeast, *Saccharomyces cerevisiae,* have yielded insights into the metabolism of this metal in humans. In yeast, iron can be captured and internalized within the cytoplasm through the multicopper oxidase Fet3 [7] and transmembrane permease Ftr1 [8,9]. The genes encoding these components, in addition to others involved in iron metabolism, constitute a regulon under the control of the transcription factor Aft1 (and its paralogue Aft2) [10,11]. The intracellular localization of Aft1 is regulated by iron availability, whereby it is localized within the nucleus when iron is scarce and translocates into the cytoplasm when iron becomes replenished [12]. The karyopherin/importin Pse1 mediates nuclear import of Aft1 in response to iron starvation [13]. Remarkably, cellular components that link iron scarcity to Aft1 nuclear localization remain poorly understood.

The TOR (target of rapamycin) is a universal sensor and integrator of nutritional signals in eukaryotes and exists in two proteins complexes, TORC1 and TORC2, where TORC1 is sensitive to the antibiotic rapamycin [14,15,16]. In yeast, TORC2 is localized at or adjacent to the plasma membrane where it phosphorylates and activates Ypk1 (or its paralogue Ypk2) [17] or Pkc1 [18]. TORC2 phosphorylates Ypk1 at two sites within a C-terminal regulatory domain [16]. Ypk1 is also phosphorylated within its kinase domain by Pkh1/Pkh2, which are homologous of mammalian PDK1 [19]. Ypk1 has a wide variety of cellular activities, including direct involvement in the biosynthesis of sphingolipids. Upon sphingolipid depletion, Ypk1 phosphorylates and inhibits both Orm1 and Orm2, which are themselves inhibitors of serine palmitoyltransferase (SPT), the enzyme that catalyzes the initial step of sphingolipid biosynthesis [20]. Treatment with Myriocin, a small-molecule inhibitor of SPT, signals to Ypk1 via TORC2 through a complex feedback activation loop [21]. Independently, Ypk1 phosphorylates Lac1 and Lag1, the two catalytic subunits of the enzyme ceramide synthase (CerS), thereby stimulating ceramide biosynthesis [22]. CerS activity also requires casein kinase 2 (CK2), which independently phosphorylates Lac1 and Lag1 and contributes to the stability of CerS and its localization within the ER [23]. Ceramides are subsequently converted in the Golgi to complex sphingolipids: inositol phosphorylceramide (IPC), mannosylinositol phosphorylceramide (MIPC), and mannosyl-diinositol phosphorylceramide [M(IP)2C] (see review [24]).

In addition to TORC2-Ypk1 and CK2, other signaling kinases have been linked to sphingolipids. For example, Sch9 kinase is a TORC1 target and has been implicated in the regulation of related of *LAG1* and *LAC1*, as well as the ceramidase genes *YPC1* and *YDC1*. Consequently, deletion of *SCH9* causes impairment of LCB levels [25]. Decreased levels of sphingolipids upon impaired TORC2/Ypk1 signaling are also associated with increased levels of reactive oxygen species (ROS) [26], which impacts mitochondrial activity and, potentially, iron homeostasis. Additional studies have reported connections between iron and sphingolipids, including a role for sphingolipids in iron toxicity [27], as well as influencing survival in the stationary phase [28].

We demonstrated previously that TORC2-Ypk1 is required as a positive regulator of autophagy that is induced when iron is limiting. In this context, the autophagy machinery is only induced when TOR2/YPK1 is active, suggesting that these proteins are required for the transmission of a signal for iron scarcity. We also demonstrated this response contributes to extend the chronological life [29].

In mammals, it has been described that TOR regulates iron homeostasis through the modulation of iron transporters and cellular iron flux [30].

In this study, we show that TORC2/Ypk1 mediates the signal for iron deprivation to Aft1. This signal is independent of ROS accumulation, mitochondrial function or changes in Pse1 localization, the nuclear transporter of Aft1. We also present evidence that nuclear accumulation of Aft1 in response to iron deprivation requires appropriate levels of sphingolipids and that this is mediated by TORC2-Ypk1.

## 2. Results

### 2.1. TORC2 and Ypk1 Are Required for Optimal Aft1 Activity during Iron Starvation 

**Figure 1:**
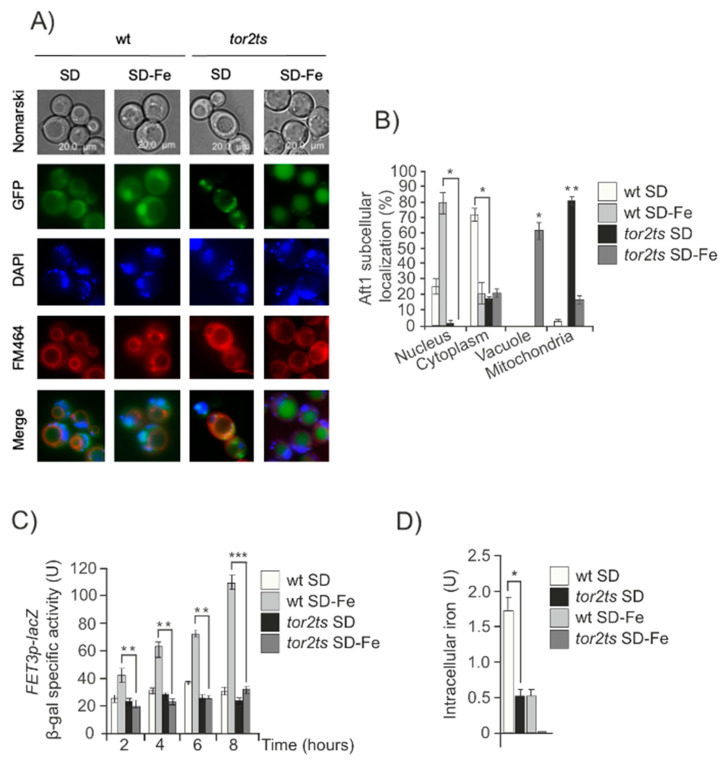
TORC2 regulates iron starvation signal. (**A**) wt and *tor2ts* cultures transformed with pAft1GFP. A preculture of exponentially growing cells at 25 °C was set. Cells from this preculture were transferred to 38 °C to OD_600_: 0.3 for 2 hours. Aliquots were collected for in vivo observation in the fluorescence microscope. (**B**) Histogram of Aft1 localization quantified in the experiment described in (**A**) was calculated upon microscopic observation of 1000 cells. The results shown are normalized and only the alive cells are taken into account. (**C**) ß-galactosidase activity content determination in wt and *tor2ts* strains. Cells were grown at 30 °C in SD media to OD_600_:0.4. Aliquots were taken, washed and transferred to SD or SD-Fe media at 38 °C for 8 hours. Samples were taken every two hours to determine the ß-galactosidase activity reporter construct. (**D**) Intracellular iron content was determined in wt and *tor2ts* exponential cultures grown in SD or SD-Fe media at 38 °C as described under Material and Methods. For all the figures: Error bars in the histograms represent the standard deviation (SD) calculated from three independent experiments. Significance of the data was determined by *p*-values from a Student unpaired *t*-test denoted as follows: * 0.05 > *p* > 0.01; ** 0.01 > *p* > 0.001; *** 0.001 > *p* > 0.0001.

In a previous study, we demonstrated a requirement for TORC2-Ypk1 signaling to induce autophagy under conditions of iron deficiency. This observation made us explore the potential connection between TORC2-Ypk1 and Aft1 during iron starvation. We first used a previously characterized temperature sensitive allele of TOR2 (*tor2ts*), an essential and central component of TORC2 [31]. We studied the role of Aft1, a transcription factor that regulates iron homeostasis, by examining its cellular localization, the expression of one of its reporter genes (*FET3*), and the levels of intracellular iron in response to varying iron concentrations. We found that when iron was deprived, in the *tor2ts* strain, Aft1 was barely present in the nuclei, with most of the protein scattered throughout the cytoplasm or concentrated in vacuoles (Figure 1A,B). This was in contrast to what was observed in the wild-type (wt) cell culture, where Aft1 rapidly translocated to the nucleus in response to low iron levels (Figure 1A,B). These findings correlated with the expression of *FET3* and iron accumulation under iron starvation conditions. In the *tor2ts* strain, both of these parameters were significantly lower than those observed in wt cells due to impaired induction of the iron regulon and internalization of iron (Figure 1C,D). We also observed the localization of Aft1 in the mitochondria in the *tor2ts* mutant under optimal growth conditions, whereas Aft1 is located in the cytoplasm in wt cells (Figure 1A,B). These results suggest that TORC2 plays a role in linking iron deficiency to the function of Aft1.

TORC2 phosphorylates and activates Ypk1, leading to many downstream signaling events, including crosstalk to Pkc1 and Sch9 kinases [32]. To map the iron starvation signal from TORC2-Ypk1 to Aft1, we analyzed the potential involvement of Pkc1/Slt2 kinases with activation that depends on TORC2. None of the mutants lacking these kinases shown any difference in Aft1 behavior as compared to the wt strain in response to iron starvation (Figure S1A). Therefore, the potential role that Pkc1/Slt2 kinases could play in Aft1 nuclear translocation upon iron starvation was ruled out.

Given that in a recent paper, we described a genetic relationship between Tor2/Ypk1 and Tor1, in the context of iron deprivation and induction of autophagy [29]. We decided to rule out the possible role of Tor1 and one of its effectors Sch9 [32] also involved in sphingolipids regulation [25], in Aft1 cellular localization when iron is scarce. Our results indicate that neither Tor1 nor Sch9 are involved in this signaling process since neither the absence of Tor1 nor rapamycin treatment affected Aft1 nuclear localization in response to iron depletion (Figure S1B). 

### 2.2. Neither Oxidative Stress, Mitochondrial Function, Iron Compartmentalization nor Pse1 Nuclear Transporter Are Signals to Drive Aft1 Localization in ypk1 Cells Depleted for Iron

**Figure 2:**
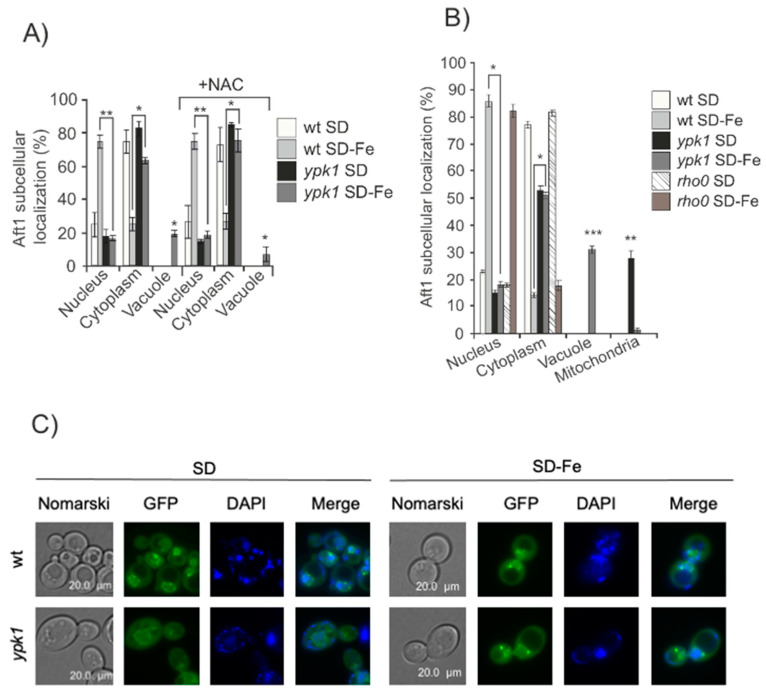
Aft1 miss localization in iron-starved *ypk1* mutant cells was not due to oxidative stress or mitochondrial dysfunction. (**A**) wt and *ypk1* cells expressing pAft1GFP were exponentially grown in SD or SD-Fe media with or without NAC overnight. Aliquots were collected for microscopic observation. The histogram represents percentages of in vivo Aft1 localization. (**B**) Aft1 subcellular localization in wt, *ypk1* and *rho0* strains grown in the same conditions as Figure 1A. (**C**) wt and *ypk1* mutant expressing pPse1GFP were grown to the log phase (OD_600_:0.6) in SD or SD-Fe medium at 30 °C. Microscopic images represent Pse1 intracellular localization. * 0.05 > *p* > 0.01; ** 0.01 > *p* > 0.001; *** 0.001 > *p* > 0.0001.

In order to check whether the observed aberrant Aft1 localization in *ypk1* mutant was an indirect consequence of either oxidative stress or mitochondrial dysfunction caused by iron deprivation [33] we added the antioxidant N-acetylcisteine (NAC) to both *ypk1* and wt cultures and observed equivalent results as those obtained in the absence of the antioxidant (Figure 2A). We also used a *rho0* strain, deficient in mitochondrial DNA, and upon iron deprivation, Aft1 localized to the nuclei as described for wild-type cells (Figure 2B). These results lead us to conclude that Aft1 miss-localization in iron-starved *ypk1* mutant cells was not a consequence of oxidative stress nor of mitochondrial dysfunction.

Pse1 is the only known transporter for Aft1 which mediates its translocation from the cytoplasm to the nucleus upon iron starvation [13]. We wondered whether in the absence of Ypk1, Pse1 was precluding Aft1 nuclear localization upon iron deprivation. Pse1 was localized to the nuclear envelope (Figure 2C), independently on the presence or absence of Ypk1, both in iron depleted cells or upon iron replenishment. In conclusion, Pse1 is not involved in Aft1 localization in *ypk1* mutant cells in response to iron starvation. 

**Figure 3:**
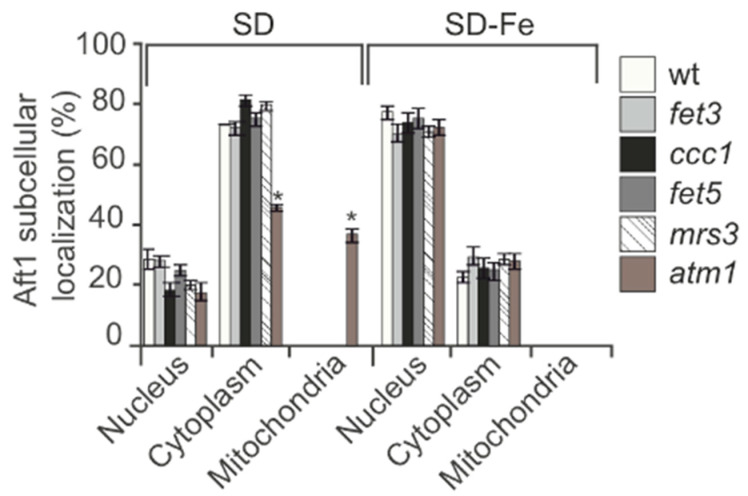
Iron accumulation in organelles do not determine Aft1 localization upon iron depletion. wt, *fet3, ccc1, fet5, mrs3* and *atm1* mutants were transformed with pAft1GFP and grown to the log phase in SD or SD-Fe media with amino acids at 30 °C. Samples were collected for in vivo observation in the fluorescence microscope. * 0.05 > *p* > 0.01.

We also considered the possibility that the signal to Aft1 nuclear localization in response to iron starvation could be originated in one of the cellular compartments where iron is stored. This would imply that one specific iron transporter would be conditioning the cellular signaling to Aft1. In order to analyze this, we made different mutants in each of the known iron transporters: Fet3: plasmatic membrane iron importer; Ccc1: vacuolar iron importer; Fet5: iron exporter from the vacuole; Mrs3: mitochondrial iron importer; Atm1: mitochondrial exporter of iron-sulfur clusters. Aft1 localization in all the mutants tested was equivalent to that determined in wt cells growing in SD-Fe (Figure 3). These observations lead us to conclude that iron accumulation in specific cellular compartments is not the main signal that determines Aft1 translocation to the nucleus when iron is limited. 

### 2.3. Lack of YPK1 Prevents Aft1 Nuclear Translocation in the Absence of Iron 

**Figure 4:**
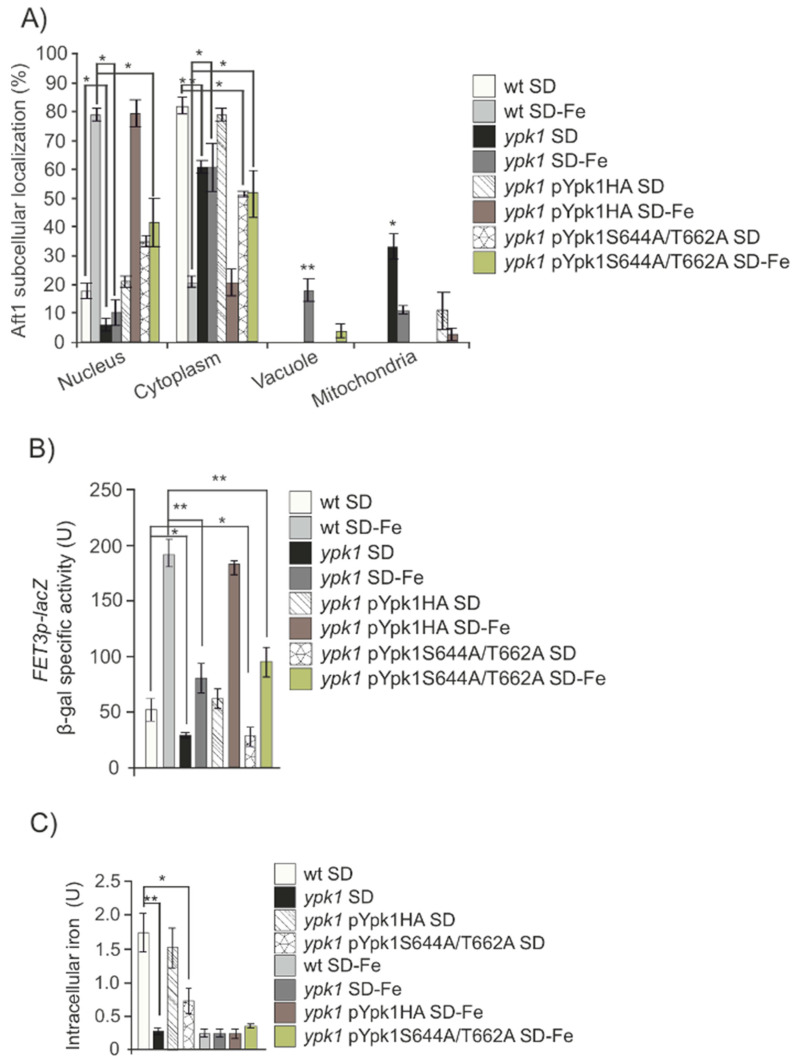

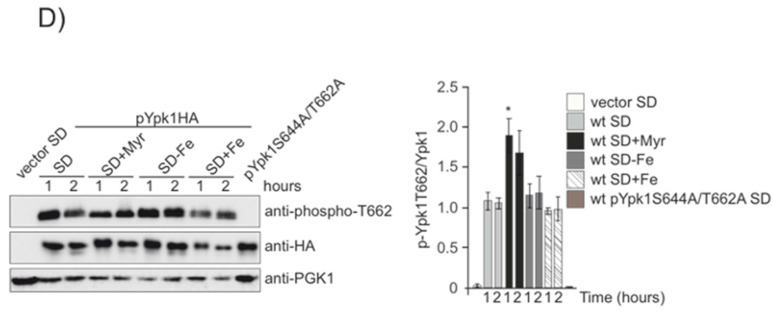
TORC2 phosphorylation to Ypk1 regulates iron starvation signal. (**A**) wt, *ypk1, ypk1*+pYpk1HA and *ypk1*+pYpk1^S644A/T662A^ transformed with the plasmid pAft1GFP, were logarithmically grown in SD medium plus amino acids or in iron-free SD (SD-Fe) to be observed in fluorescence microscopy. The histogram represents percentages of in vivo nuclear, cytoplasmic, vacuolar or mitochondrial localization out of 1000 cells. (**B**) Strains wt, *ypk1, ypk1*+pYpk1HA and *ypk1*+pYpk1^S644A/T662A^ were each transformed with plasmid p*FET3*-LacZ. Cells were grown at 30 °C in SD media overnight to OD_600_:0.6. (**C**) Intracellular iron content was determined in wt, *ypk1, ypk1*+pYpk1HA and *ypk1*+pYpk1^S644A/T662A^ exponential cultures grown in SD or SD-Fe media as described under Material and Methods. (**D**) wt strain was transformed with pC-terminal3-HA, pYpk1HA or pYpk1^S644A/T662A^. Cells were grown in SD to the exponential phase, the cultures were washed and transferred to SD, SD+Myr, SD-Fe or SD+Fe samples were collected at indicated times for Western blot analysis, probing with anti-phospho-T662, anti-HA and anti-PGK1 antibodies. The histogram represents the ratio between Ypk1 phospho-T662 and total Ypk1. * 0.05 > *p* > 0.01; ** 0.01 > *p* > 0.001.

Ypk1 is phosphorylated and regulated by TORC2 in two specific residues (see review [32]). We wondered whether Ypk1 was involved in the process of sensing iron starvation downstream of Tor2. Upon iron depletion, we could observe a similar but less dramatic phenotype than that shown above for *tor2ts* mutant (Figure 1A–D). Aft1 mostly remained in the cytoplasm or partly in the vacuole, (Figure 4A). This localization correlated to the detection of both a very low iron intracellular content and significantly reduced *FET3* expression in iron deprived *ypk1* cultures, as compared to wt cultures (Figure 4B,C). 

In order to discard any artifact caused by the mechanical process of *ypk1* construct, we decided to check the specificity of *YPK1* function in the response to iron starvation by means of a complementation assay. We transformed *ypk1* mutant cells with a plasmid overexpressing Ypk1 and observed a clear complementation given that Aft1 both localization and function were equivalent to that observed in wt cells and confirming the hypothesis that Ypk1 plays a direct role in the cellular response to iron deprivation (Figure 4A–C). In order to observe the actual localization of the recently synthetized protein, we blocked protein synthesis in both wt and *ypk1* strains with cycloheximide. This assay reinforced our former hypothesis, since we obtained identical results regarding Aft1 localization in both wt and *ypk1* strains (Figure 4A), and also allowed us to conclude that Ypk1 was not involved in iron replenishment since in this condition Aft1 translocated from the nucleus to the cytoplasm in both wt and *ypk1* cultures (Figure S2). Given the relevance of our results, we validated them in a different genetic background (Figure S3). Therefore, our results suggest that in conditions of iron starvation, the absence of Ypk1 precludes Aft1 translocation to the nucleus and consequently seriously impairs the induction of the iron regulon in response to the low levels of the metal. 

Tor2 activates Ypk1 upon phosphorylation in two residues: S644 and T662 [16,20]. In order to biochemically characterize this function, we made use of an antibody that specifically recognizes both phosphorylated residues and analyzed Ypk1 phosphorylation Tor2 specific in wild-type cells starved or not for iron. We did not detect significant differences (Figure 4D) being Ypk1 constitutively phosphorylated by Tor2 during exponential growth in both conditions. Our results strongly suggest that cells actually require a constitutively activated Tor2-Ypk1 pathway in order to signal Aft1 correct response to iron availability. In order to gain further insight into this mechanism, we used a complementation approach by transforming *ypk1* mutant with a plasmid bearing the wild-type *YPK1* coding sequence and another plasmid mutated in both S644A/T662A *YPK1* residues specifically phosphorylated by TORC2. Upon analyzing the results, we noted that whereas the plasmid bearing wild-type *YPK1* ORF completely complemented *ypk1* strain, regarding Aft1 function in response to iron deprivation, the plasmid carrying pYpk1^S644A/T662A^, was not able to complement a *ypk1* mutant under the above mentioned nutritional conditions (Figure 4A–C). Our results strongly suggest that Tor2 signals iron scarcity through Ypk1 to Aft1 function. 

### 2.4. Complex LCBs Levels Control Aft1 Nuclear Localization and Function When Iron Is Limited

**Figure 5:**
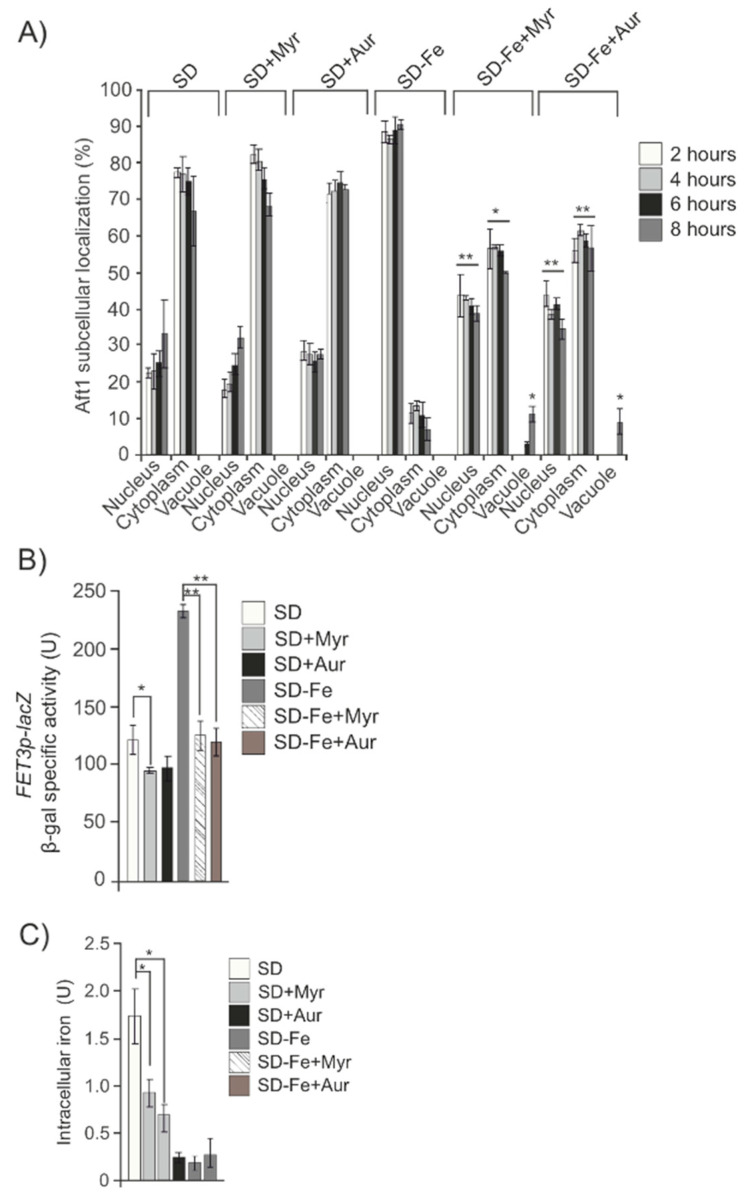

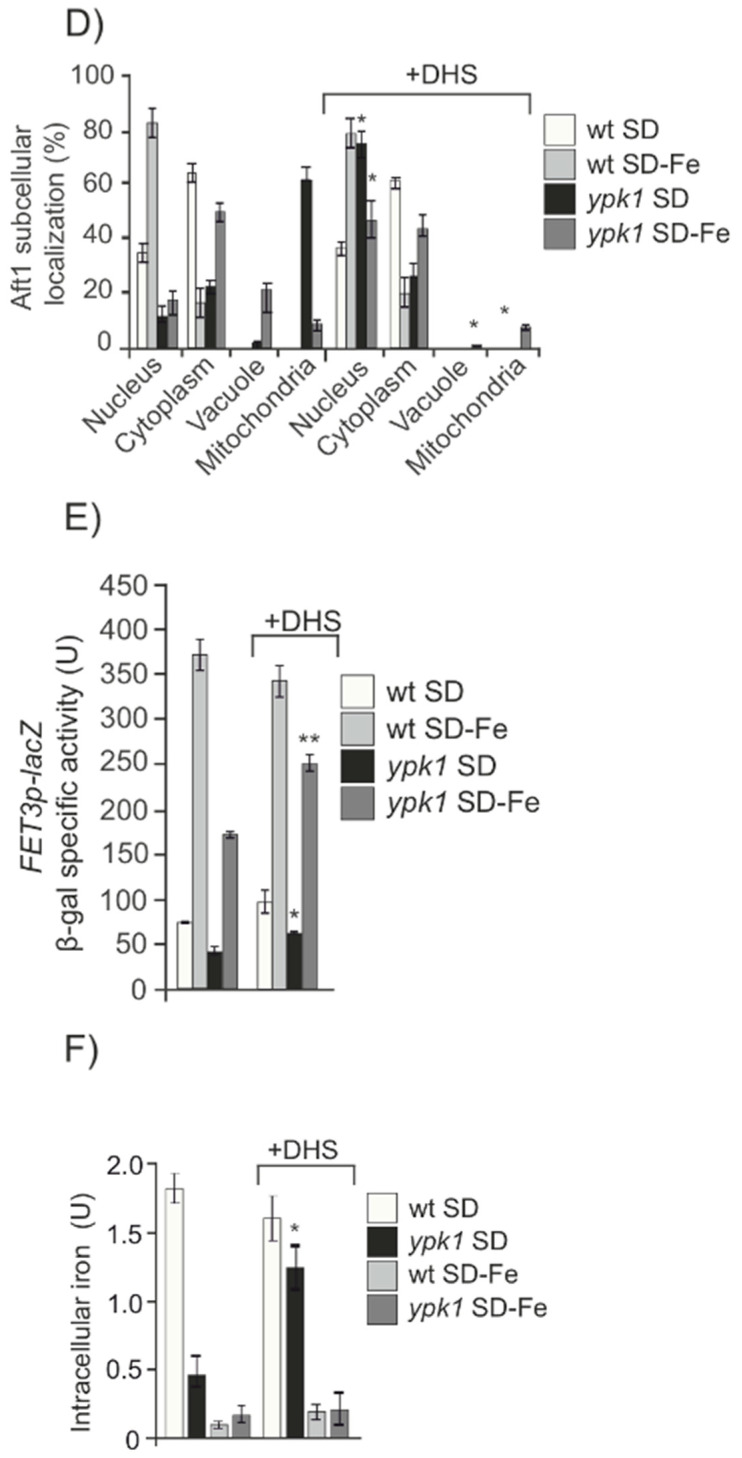
Levels of sphingolipids modify Aft1 localization. (**A**) wt strain transformed with pAft1GFP was grown in SD or SD-Fe medium with amino acids and treated or not with Myriocin (Myr) or Aureobasidin A (Aur) samples were taken at indicated times to analyze Aft1 subcellular localization (**B**) wt bearing p*FET3*-LacZ was grown in SD or SD-Fe medium with amino acids and treated or not with Myriocin or Aureobasidin A. Samples were taken at 8 h. (**C**) Intracellular iron content in a wt strain grown in SD or SD-Fe treated or not with Myriocin or Aureobasidin A until the logarithmic phase. (**D**–**F**) Aft1 subcellular localization, ß-galactosidase activity and intracellular iron content, respectively, in wt and *ypk1* strains grown exponentially in SD or SD-Fe and treated or not with DHS. * 0.05 > *p* > 0.01; ** 0.01 > *p* > 0.001.

Ypk1 kinase activity controls sphingolipids homeostasis upon TORC2 regulation. We decided to investigate downstream members of the pathway with the aim to identify at which level of the sphingolipid pathway the signal diverges to regulate Aft1 localization in response to iron concentration. Hence, we treated wild-type iron depleted cultures with Myriocin, which is a very potent inhibitor of serine palmitoyltransferase, that catalyzes the conversion into 3-ketodihydrosphingosine, the first step in sphingosine biosynthesis. We observed a high proportion of Aft1 in the cytoplasm and vacuole correlated to a significant descent both in *FET3* expression and iron content (Figure 5A–C). 

Downstream in the sphingolipids pathway, dihydrosphingosine (DHS), is a ceramide precursor, since it can be converted to ceramide by ceramide synthase (CerS), which catalyzes the formation of an amide bond between the LCB and a C26 very long-chain fatty acid [24].

CerS activity is regulated by direct phosphorylation of the catalytic subunits, Lac1 and Lag1, through TORC1, TORC2, and casein kinase 2 [22,23]. We observed that blocking CerS activity upon deletion of either *LAC1* or *LAG1* genes*,* or their regulatory protein CK2, significantly impaired Aft1 function upon iron starvation, since a clear descent in Fet3 expression and in Aft1 nuclear localization were detected as compared to wild-type values (Figure S4). These results were similar to those previously described in cells treated with Myriocin (Figure 5A–C).

In order to test the importance of ceramide synthesis in the process of iron deficiency signaling, we added DHS to both wt and *ypk1* iron depleted cultures and allowed them grown over night to the exponential phase. Interestingly, we could observe that addition of the precursor of ceramide to *ypk1* cultures partly restored Aft1 wild-type response to iron starvation, since 35% of the cells contained Aft1 in the nucleus correlated to a significant increase in both *FET3* expression and cellular iron content, as compared to *ypk1* cultures not added for DHS (Figure 5D–F). 

**Figure 6:**
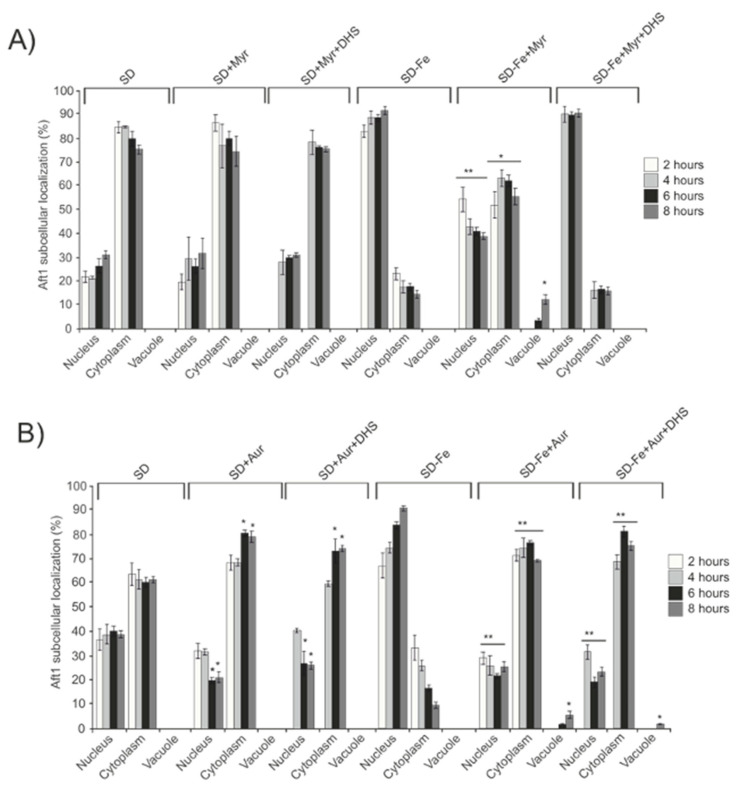
Complex sphingolipids signal Aft1 to localize to the nucleus upon iron depletion. wt cells expressing pAft1GFP were grown to OD_600_:0.4 in SD medium at 30 °C. (**A**) Aliquots were taken, washed and transferred to either SD, SD+Myr, SD-Fe or SD-Fe+Myr media. After 2 hours, the cultures with Myriocin (Myr) were divided in two halves and DHS was only added to one of them, the other half was maintained as a control. Samples were collected for in vivo observation in the fluorescence microscope, every two hours for eight hours. (**B**) Aliquots were taken, washed and transferred to SD, SD+Aur, SD-Fe or SD-Fe+Aur media. After 2 hours the cultures with Aureobasidin A (Aur) were divided into two halves and DHS was only added to one of them. Samples were collected for in vivo observation in the fluorescence microscope at the indicated times. * 0.05 > *p* > 0.01; ** 0.01 > *p* > 0.001.

Aureobasidin A is a cyclic depsipeptide which inhibits the inositolphosphorylceramide synthase, *AUR1,* downstream of ceramide synthesis, thus preventing the accumulation of long-chain complex sphingolipids. Addition of Aureobasidin A to cultures depleted for iron also precluded Aft1 translocation into the nucleus and consequently negatively affected both *FET3* induction and iron intracellular accumulation (Figure 5A–C). Next, we also added DHS to wild-type cells blocked in sphingolipid synthesis after a previous treatment with Myriocin. In this case and similarly to that described above in *ypk1* cultures, we observed that DHS was able to partly restore Aft1 function in response to iron starvation (Figure 6A). However, and contrary to the results recently described, DHS addition to iron deprived wt cultures treated with Aureobasidin A, did not restore Aft1 wild-type function in response to iron starvation (Figure 6B). Taking altogether these results we conclude that the activity of the sphingolipid pathway leaded by TORC2 and *YPK1* to induce the synthesis of long-chain sphingolipids, is the main signal converging in Aft1 and induces its nuclear translocation to activate the iron regulon in order to maintain iron homeostasis in response to iron deficiency. 

## 3. Discussion

Iron is an essential metal for living organisms [34]. However, its excess can cause dramatic damage to cells mainly through oxidative reactions [35], whereas its deficiency is associated with several metabolic disorders [36], consequently, iron homeostasis must be tightly regulated in all living cells, part to hinder the potential damage that its dysregulation can provoke. In *Saccharomyces cerevisiae,* Aft1 is the main responsible for iron utilization and homeostasis. Its function has been associated with its transcriptional regulation correlated to its nuclear localization in order to maintain the correct equilibrium of the iron cellular requirements [11,31,37]. Grx3/Grx4 both regulate the translocation of Aft1 from the nucleus to the cytoplasm when iron is replenished [31]. However, when iron is depleted from the culture medium, Aft1 translocation from the cytoplasm to the nucleus has not been associated with any accompanying protein to date. Recently, we demonstrated that iron deprivation induces bulk autophagy in a manner completely dependent on Ypk1 and Tor1 [29]. Our next interest was to explore the potential connection between the iron homeostasis regulator Aft1 and Tor2/Ypk1 in the cellular response to iron starvation. Here, we show evidence demonstrating that Aft1 translocation to the nucleus in response to iron deprivation is dependent on TORC2/Ypk1 signaling pathway. Interestingly, preliminary and unpublished data from J. Thorner [16] support the conclusion that TORC2-Ypk1 signaling could be related to iron metabolism at least mechanistically. It has been widely demonstrated that TORC2 through Ypk1, positively signals the activation of the sphingolipid pathway in conditions unrestricted of nutrients [32]. Here, we demonstrate that iron deprivation determines Aft1 nuclear localization in a manner dependent on the activity of the sphingolipid synthesis through TORC2/Ypk1 signaling activity.

We also present evidence demonstrating that Aft1 miss-localization upon iron starvation, in a context of sphingolipid synthesis impairment, is not caused by a deficient expression of the nuclear import receptor Pse1, which remains localized in in the nuclear membrane regardless iron availability. Apart from Ypk1, Pkc1 is another target of TORC2 in the response to certain types of stress [18]. Our results clearly show that Pkc1 does not participate in the regulation of Aft1 nuclear translocation and the consequent induction of the iron regulon upon iron deprivation. 

Sphingolipids are essential components of the cellular membranes that frequently participate in several signaling processes in all the eukaryotic cellular systems [27]. In line with this, we explored the possibility that low iron concentration localized in specific cellular compartments could be the signal to direct Aft1 into the nucleus. Our results made us rule out this hypothesis since all the mutants in the various iron-dependent membrane transporters checked in this study presented a wild-type Aft1 response upon iron deprivation.

Iron can promote the production of ceramides in humans [38] as previously shown in *Saccharomyces cerevisiae* [27]. The connection between sphingolipids and iron has also been reported [39] upon the demonstration that iron deprivation caused the induction of some sphingolipids. Other authors have described a relationship between ceramides and the modulation of iron levels [40]. In our study, we observe that sphingolipids are constitutively activated in wild-type cells growing exponentially in conditions not limited for nutrients, independently of iron availability. As long as the sphingolipid pathway is active, iron depletion signal is correctly transmitted to Aft1, resulting in its translocation to the nucleus and the consequent induction of the iron regulon. A blockade in long-chain sphingolipids synthesis interrupts this signal and provokes a cytoplasmic/vacuolar Aft1 localization when iron is not available. This aberrant localization is detrimental for survival since forcing nuclear localization of Aft1 by using the pAft1C291F allele in a *ypk1* mutant significantly increased cell survival close to wild-type levels (Figure S5), which clearly remarks the importance that sphingolipids signaling has in the response to iron deprivation.

In humans, the mTOR pathway can regulate the expression of CD71, which is responsible for cellular iron uptake [41]. Indeed, it has been recently proposed that inhibition of mTOR could reduce iron accumulation and thus, lessens the neurodegenerative effects induced by this metal [41]. These observations are in accordance with our results in that Tor2 inhibition negatively converges in Aft1 reducing the expression of the iron regulon. Fet3 is the responsible for extracellular iron uptake in budding yeast and because is included in the iron regulon, its expression is drastically diminished upon Tor2 inhibition and the subsequent blockade of the sphingolipid pathway. 

We have analyzed the sphingolipid pathway at various levels: i) upstream by blocking the kinase Ypk1, or using Myriocin which blocks the activity of the palmitoiltranspherase and consequently the production of sphingosine, and ii) downstream in the signaling cascade, by inhibiting both ceramide and complex sphingolipids synthesis. Administration of DHS which promotes ceramides and consequently complex sphingolipids synthesis complements the lack of signaling to Aft1 in iron-starved cells when Tor2/Ypk1 are mutated (Figure 5D–F). 

In this study, we show evidence demonstrating that long-chain sphingolipids play a crucial role in the transmission of the iron starvation signal to Aft1, since addition of the precursor of ceramides (DHS), did not restore Aft1 wild-type response to iron deprivation in cells blocked in the production of long-chain sphingolipids, upon Aureobasidin A treatment. 

Several studies have demonstrated that Ypk1 is related to ROS production in a manner dependent on sphingolipids [26]. Our findings rule out the possibility that sphingolipid downregulation caused by *YPK1* deletion in iron deprivation conditions, impinges on Aft1 wild-type response in a manner dependent on ROS production or mitochondrial function.

Recently, Jordà et al. [40] have published that sterol synthesis impairment precludes Aft1 function. Although Ypk1 has been suggested to be an ergosterol sensor [42], subsequent studies discarded this connection and demonstrated the independence between ergosterol and Ypk1 activity [20]. 

Our results offer a demonstration of the importance of the sphingolipid pathway mediated by TORC2/Ypk1 proteins in the cellular response to iron scarcity in the context of iron homeostasis. In addition, our findings suggest that Aft1 not only regulates the expression of a group of genes needed for iron acquisition, detoxification or storage but also participates in remodeling the metabolism as previously proposed for different metalosensors in the eukaryotic model *Saccharomyces cerevisiae* [43]. Further studies will be required in order to ascertain the molecular mechanism involved in Aft1 regulation by long-chain sphingolipids as well as its potential role in vacuolar function.

## 4. Material and Methods

### 4.1. Yeast Strains and Plasmids

*Saccharomyces cerevisiae* strains used in this study are listed in Table 1. New mutants described in this work were obtained by one-step disruption method that uses the NatMx4 or KanMx4 cassettes [29]. Strain GSL421 was constructed upon the integration of plasmid pAft1C291F-HA previously digested with EcoRV. GSL451 strain was constructed upon the integration of pYpk1^S644A/T662A^ plasmid previously digested by BstEII. Strains GSL454 and GSL455 were constructed upon the integration of plasmid pYpk1-HA previously digested with BstEII. The plasmid pYpk1-HA was obtained upon Ypk1 cloning into the PmeI and NotI sites of the integrative vector pMM351. The plasmid pPse1GFP was obtained through Pse1 cloning into the BamHI and SalI sites of the pUG35 plasmid.

Plasmid descriptions are listed in Table 2. Each particular ORF was amplified by PCR from genomic DNA and cloned in the specific plasmid. 

### 4.2. Growth Conditions and Reagents

Yeasts were grown at 30 °C in SD medium (2% glucose, 0.67% yeast nitrogen base that lacked the corresponding amino acids for plasmid maintenance) plus amino acids [54]. For iron depletion conditions (SD-Fe), SD medium was used with a yeast nitrogen base that is free of iron plus the addition of 80 µM of 4,7-diphenyl-1,10-phenanthrolinedisulfonic acid (BPS) (Sigma, 146617, St. Louis, MO, USA). 

We present a list of reagents detailing the final concentration in culture media and from which company they were purchased: N-acetylcysteine (NAC) 5 mM (Sigma, A9165); rapamycin (Rapa) 200 ng/mL (Sigma, R0395); cycloheximide (CHX) 150 mg/mL (Sigma, C4859); DAPI 2 mg/mL (Sigma, D9541); (N-(3-triethylammoniumpropyl)-4-(p-diethylaminophenylhexatrienyl)) pyridinium dibromide (FM4-64) 30 µg/µL (Invitrogen, T-3166); Myriocin (Myr) 2 mM (Sigma, M1177); D-erythro-Dihydrosphingosine (DHS) 20 µM (Sigma, D3314); Aureobasidin A (Aur) 250 ng/mL (MedChem Express, HY-P1975, South Brunswick Township, NJ, USA). Cell cultures were exponentially grown at 600 nm [OD_600_] of 0.6 or longer times as indicated. Iron was added as ammonium iron (III) sulfate hexacahydrate [NH_4_Fe(SO_4_)_2_•6H_2_O] (+Fe) (Sigma, F1543) at a final concentration of 10 mM.

### 4.3. Endogenous Iron Measurements

Endogenous iron measurements were performed according to the colorimetric assay described in [53].

### 4.4. ß-Galactosidase Activity

ß-galactosidase activity was determined according to [55], with some variations. A volume of 1 mL of cell culture at [O.D._600_] of 0.6 was centrifuged, and the pellets were suspended in 50 µL buffer Z (Na_2_HPO_4_ 60 mM (Serva, 30200.01, Catoosa, OK, USA); NaH_2_PO_4_ 40 mM (Serva, 13472-35-0); KCl 10 mM (Serva, 26868); MgSO_4_ 1 mM (Sigma, M7634-100G); ß-mercaptoethanol 50 mM (BioRad, 161-0710, Hercules, CA, USA)) plus 2.5 µL sarcosyl 10% (Sigma, T4376) and 0.5 µL toluene (Merck, 244511, Rahway, NJ, USA). After that, 150 µL of buffer Z and 50 µL *o*-nitrophenyl-ß-D-galactopyranoside (ONPG) 4 mg/mL (Sigma, N1127) were added and subsequently incubated at 28 °C for 5 min. Finally, 500 µL Na_2_CO_3_ 1 M (Sigma, S7795-500G) was added to stop the reaction. Absorbance was measured at 420 nm. 

### 4.5. Protein Extraction and Immunoblot Analyses

We follow an identical procedure as described in [29]. Total yeast protein extracts were prepared as previously described in [29]. The antibodies for Western blotting were as follows: anti-HA 3F10 (Roche Applied Science, 12158167001, Penzberg, Germany), was used at a dilution of 1: 2000 in 0.25% non-fat milk and the corresponding secondary was goat anti-Rat IgG horseradish peroxidase conjugate (Millipore, AP136P, Burlington, MA, USA). Anti-phospho-glycerate kinase 1 (anti-PGK-1) (Invitrogen, 459250, Waltham, MA, USA) was used at a dilution 1: 1200, with the secondary antibody anti-Mouse horseradish peroxidase conjugate (GE Healthcare, LNA931v/AG, Chicago, IL, USA). Anti-phospho-Ypk1 (T662) (from Dr. Ted Powers) at a dilution of 1:20,000, with the secondary antibody anti-Rabbit horseradish peroxidase conjugate (GE Healthcare, LNA934v/AG). They were used as indicated by the manufacturers.

The protein–antibody complexes were visualized by enhanced chemiluminescence, using the Supersignal substrate (Pierce, 34577, Appleton, WI, USA) in a Chemidoc (Roche Applied Science). 

For all the figures: We used anti-PGK1 to detect PGK1, selected as loading control in the Western blots shown in this study. For Western blot in this paper, we have selected representative samples. 

### 4.6. Fluorescence Microscopy

Cells were visualized under the fluorescence microscope (Olympus BX-51) using 60X magnification. Cellular localizations were registered at the times indicated in the text under specific described conditions. 

### 4.7. Statistical Analysis

We followed the same procedure as described in [29]. Error bars in the histograms represent the standard deviation (SD) calculated from three independent experiments. Significance of the data was determined by *P-*values from a Student unpaired *t*-test denoted as follows; * 0.05 > *p* > 0.01; ** 0.01 > *p* > 0.001; *** 0.001 > *p* > 0.0001; **** *p* > 0.0001.

## Figures and Tables

**Table 1:** Yeast strains.

Strain	Genotype	Source
CML128	*MATa leu2-3,112, ura3-52, trp1, his4*	[44]
GSL034	CML128 background, *tor1::KanMx4*	[45]
GSL190	CML128 background, *slt2::KanMx4*	[46]
GSL205	CML128 background, *sch9::NatMx4*	[46]
GSL280	CML128 background, *tetO_7_AFT1C291F-HA::LEU2*	[47]
GSL308	CML128 background, *tetO_7_Aft1-HA::LEU2*	[47]
GSL384	CML128 background, *ypk1::KanMx4*	[29]
GSL385	CML128 background, *ypk1::KanMx4 tetO_7_Aft1-HA::LEU2*	[47]
GSL410	CML128 background, *pkc1::LEU2*	[48]
GSL420	CML128 background, *ypk1::KanMx4 atg7::NatMx4*	This work
GSL421	CML128 background, *ypk1::KanMx4 tetO_7_AFT1C291F-HA::LEU2*	This work
GSL430	CML128 background, *lac1::KanMx4*	This work
GSL431	CML128 background, *lag1::KanMx4*	This work
GSL435	CML128 background, *fet3::KanMx4*	This work
GSL436	CML128 background, *ccc1::KanMx4*	This work
GSL437	CML128 background, *mrs3::KanMx4*	This work
GSL447	CML128 background, *fet5::KanMx4*	This work
GSL448	CML128 background, *atm1::KanMx4*	This work
GSL451	CML128 background, *ypk1::KanMx4 YPK1S644AT662A-HA::LEU2*	This work
GSL454	CML128 background, *Ypk1-HA::LEU2*	This work
GSL455	CML128 background, *ypk1::KanMx4 Ypk1-HA::LEU2*	This work
R43	CML128 background, *rho0*	This work
BY4741	*MATa his3-1, leu2, met15, ura3*	[49]
GSL404	BY4741 background, *ypk1::KanMx4*	This work
W303	*MATa ade2-1, trp1-1, leu2-3,2-111, his3-11,75, ura3*	[50]
GSL417	W303 background, *tor2ts::LEU2*	[31]
LHY291	*MATa his3, trp1, lys2, ura3, leu2, bar1*	[23]
PLY979	LHY291 background, *cka2::TRP1*	[23]

**Table 2:** Plasmids employed.

Plasmid	Marker	Promoter	Epitope	Source
pAft1-GFP	*URA3*	*MET25*	GFP	[51]
ptetO_7_Aft1-HA	*LEU2*	*tetO_7_*	HA	[51]
pAft1C291F-HA	*LEU2*	*tetO_7_*	HA	[47]
pFet3-LacZ	*URA3*	*FET3*		[52]
pUG35	*URA3*	*MET25*	GFP	[53]
pPse1-GFP	*URA3*	*MET25*	GFP	This work
pC-terminal3-HA	*URA3*	*MET25*	HA	[50]
pMM351	*LEU2*	*ADH1*	HA	[29]
pYpk1-HA	*LEU2*	*ADH1*	HA	This work
pYpk1^S644A/T662A^	*LEU2*	*ADH1*	HA	[50]

## Supplementary Materials

The following supporting information can be downloaded at: https://www.mdpi.com/article/10.3390/ijms24032438/s1.

## References

[1] Outten C.E., Albetel A.-N. (2013). Iron sensing and regulation in *Saccharomyces cerevisiae*: Ironing out the mechanistic details. Curr. Opin. Microbiol..

[2] Bogdan A.R., Miyazawa M., Hashimoto K., Tsuji Y. (2016). Regulators of Iron Homeostasis: New Players in Metabolism, Cell Death, and Disease. Trends Biochem. Sci..

[3] Eid R., Arab N.T., Greenwood M.T. (2017). Iron mediated toxicity and programmed cell death: A review and a re-examination of existing paradigms. Biochim. Biophys. Acta (BBA) Mol. Cell Res..

[4] Ramos-Alonso L., Romero A.M., Martínez-Pastor M.T., Puig S. (2020). Iron Regulatory Mechanisms in *Saccharomyces cerevisiae*. Front. Microbiol..

[5] Carmona-Gutierrez D., Bauer M.A., Zimmermann A., Aguilera A., Austriaco N., Ayscough K., Balzan R., Bar-Nun S., Barrientos A., Belenky P. (2018). Guidelines and recommen-dations on yeast cell death nomenclature. Microb. Cell.

[6] Percy L., Mansour D., Fraser I. (2017). Iron deficiency and iron deficiency anaemia in women. Best Pract. Res. Clin. Obstet. Gynaecol..

[7] Askwith C., Eide D., Van Ho A., Bernard P.S., Li L., Davis-Kaplan S., Sipe D.M., Kaplan J. (1994). The FET3 gene of *S. cerevisiae* encodes a multicopper oxidase required for ferrous iron uptake. Cell.

[8] Stearman R., Yuan D.S., Yamaguchi-Iwai Y., Klausner R.D., Dancis A. (1996). A Permease-Oxidase Complex Involved in High-Affinity Iron Uptake in Yeast. Science.

[9] Dancis A. (1998). Genetic analysis of iron uptake in the yeast *Saccharomyces cerevisiae*. J. Pediatr..

[10] Shakoury-Elizeh M., Tiedeman J., Rashford J., Ferea T., Demeter J., Garcia E., Rolfes R., Brown A.J., Botstein D., Philpott C.C. (2004). Transcriptional Remodeling in Response to Iron Deprivation in *Saccharomyces cerevisiae*. Mol. Biol. Cell.

[11] Yamaguchi-Iwai Y., Stearman R., Dancis A., Klausner R.D. (1996). Iron-regulated DNA binding by the AFT1 protein controls the iron regulon in yeast. EMBO J..

[12] Ueta R., Fujiwara N., Iwai K., Yamaguchi-Iwai Y. (2007). Mechanism Underlying the Iron-dependent Nuclear Export of the Iron-responsive Transcription Factor Aft1p in *Saccharomyces cerevisiae*. Mol. Biol. Cell.

[13] Ueta R., Fukunaka A., Yamaguchi-Iwai Y. (2003). Pse1p Mediates the Nuclear Import of the Iron-responsive Transcription Factor Aft1p in *Saccharomyces cerevisiae*. J. Biol. Chem..

[14] Saxton R.A., Sabatini D.M. (2017). mTOR Signaling in Growth, Metabolism, and Disease. Cell.

[15] González A., Hall M.N. (2017). Nutrient sensing and TOR signaling in yeast and mammals. EMBO J..

[16] Leskoske K.L., Roelants F.M., Marshall M.N.M., Hill J.M., Thorner J. (2017). The Stress-Sensing TORC2 Complex Activates Yeast AGC-Family Protein Kinase Ypk1 at Multiple Novel Sites. Genetics.

[17] Chen P., Lee K.S., Levin D.E. (1993). A pair of putative protein kinase genes (YPK1 and YPK2) is required for cell growth in *Saccharomyces cerevisiae*. Mol. Gen. Genet..

[18] Levin D.E., Fields F., Kunisawa R., Bishop J., Thorner J. (1990). A candidate protein kinase C gene, PKC1, is required for the S. cerevisiae cell cycle. Cell.

[19] Casamayor A., Torrance P.D., Kobayashi T., Thorner J., Alessi D. (1999). Functional counterparts of mammalian protein kinases PDK1 and SGK in budding yeast. Curr. Biol..

[20] Roelants F.M., Breslow D.K., Muir A., Weissman J.S., Thorner J. (2011). Protein kinase Ypk1 phosphorylates regulatory proteins Orm1 and Orm2 to control sphingolipid homeostasis in *Saccharomyces cerevisiae*. Proc. Natl. Acad. Sci. USA.

[21] Berchtold D., Piccolis M., Chiaruttini N., Riezman I., Riezman H., Roux A., Walther T.C., Loewith R. (2012). Plasma membrane stress induces relocalization of Slm proteins and activation of TORC2 to promote sphingolipid synthesis. Nat. Cell Biol..

[22] Muir A., Ramachandran S., Roelants F.M., Timmons G., Thorner J. (2014). TORC2-dependent protein kinase Ypk1 phosphorylates ceramide synthase to stimulate synthesis of complex sphingolipids. Elife.

[23] Fresques T., Niles B., Aronova S., Mogri H., Rakhshandehroo T., Powers T. (2015). Regulation of Ceramide Synthase by Casein Kinase 2-dependent Phosphorylation in *Saccharomyces cerevisiae*. J. Biol. Chem..

[24] Megyeri M., Riezman H., Schuldiner M., Futerman A.H. (2016). Making Sense of the Yeast Sphingolipid Pathway. J. Mol. Biol..

[25] Swinnen E., Wilms T., Idkowiak-Baldys J., Smets B., De Snijder P., Accardo S., Ghillebert R., Thevissen K., Cammue B., De Vos D. (2014). The protein kinase Sch9 is a key regulator of sphingolipid metabolism in *Saccharomyces cerevisiae*. Mol. Biol. Cell.

[26] Niles B.J., Joslin A.C., Fresques T., Powers T. (2014). TOR Complex 2-Ypk1 Signaling Maintains Sphingolipid Homeostasis by Sensing and Regulating ROS Accumulation. Cell Rep..

[27] Lee Y.-J., Huang X., Kropat J., Henras A., Merchant S.S., Dickson R.C., Chanfreau G.F. (2012). Sphingolipid Signaling Mediates Iron Toxicity. Cell Metab..

[28] Lester R.L., Withers B.R., Schultz M.A., Dickson R.C. (2013). Iron, glucose and intrinsic factors alter sphingolipid composition as yeast cells enter stationary phase. Biochim. Biophys. Acta (BBA) Mol. Cell Biol. Lipids.

[29] Montella-Manuel S., Pujol-Carrion N., Mechoud M.A., de la Torre-Ruiz M.A. (2021). Bulk autophagy induction and life extension is achieved when iron is the only limited nutrient in *Saccharomyces cerevisiae*. Biochem. J..

[30] Bayeva M., Khechaduri A., Puig S., Chang H.-C., Patial S., Blackshear P.J., Ardehali H. (2012). mTOR Regulates Cellular Iron Homeostasis through Tristetraprolin. Cell Metab..

[31] Pujol-Carrion N., Belli G., Herrero E., Nogues A., de la Torre-Ruiz M.A. (2006). Glutaredoxins Grx3 and Grx4 regulate nuclear localisation of Aft1 and the oxidative stress response in *Saccharomyces cerevisiae*. J. Cell Sci..

[32] Kaeberlein M., Powers R.W., Steffen K.K., Westman E.A., Hu D., Dang N., Kerr E.O., Kirkland K.T., Fields S., Kennedy B.K. (2005). Regulation of Yeast Replicative Life Span by TOR and Sch9 in Response to Nutrients. Science.

[33] Roelants F.M., Leskoske K.L., Marshall M.N.M., Locke M.N., Thorner J. (2017). The TORC2-Dependent Signaling Network in the Yeast *Saccharomyces cerevisiae*. Biomolecules.

[34] Milto I.V., Suhodolo I.V., Prokopieva V.D., Klimenteva T.K. (2016). Molecular and Cellular Bases of Iron Metabolism in Humans. Biochemistry.

[35] Toledano M.B., Delaunay A., Biteau B., Spector D., Azevedo D. (2003). Oxidative Stress Responses in Yeast. Yeast Stress Responses.

[36] Wallace D.F. (2016). The Regulation of Iron Absorption and Homeostasis. Clin. Biochem. Rev..

[37] Ueta R., Fujiwara N., Iwai K., Yamaguchi-Iwai Y. (2012). Iron-Induced Dissociation of the Aft1p Transcriptional Regulator from Target Gene Promoters Is an Initial Event in Iron-Dependent Gene Suppression. Mol. Cell Biol..

[38] Lane D.J.R., Merlot A.M., Huang M.L.H., Bae D.H., Jansson P.J., Sahni S., Kalinowski D.S., Richardson D.R. (2015). Cellular iron uptake, trafficking and metabolism: Key molecules and mechanisms and their roles in disease. Biochim. Biophys. Acta (BBA) Mol. Cell Res..

[39] Shakoury-Elizeh M., Protchenko O., Berger A., Cox J., Gable K., Dunn T.M., Prinz W.A., Bard M., Philpott C. (2010). Metabolic response to iron deficiency in *Saccharomyces cerevisiae*. J. Biol. Chem..

[40] Almeida T., Marques M., Mojzita D., Amorim M.A., Silva R.D., Almeida B., Rodrigues P., Ludovico P., Hohmann S., Moradas-Ferreira P. (2008). Isc1p Plays a Key Role in Hydrogen Peroxide Resistance and Chronological Lifespan through Modulation of Iron Levels and Apoptosis. Mol. Biol. Cell.

[41] Jodeiri Farshbaf M., Ghaedi K. (2016). Does any drug to treat cancer target mTOR and iron hemostasis in neurodegenerative disorders?. BioMetals.

[42] Li X., Gianoulis T.A., Yip K.Y., Gerstein M., Snyder M. (2010). Extensive In Vivo Metabolite-Protein Interactions Revealed by Large-Scale Systematic Analyses. Cell.

[43] Bird A.J. (2008). Metallosensors, the ups and downs of gene regulation. Adv. Microb. Physiol..

[44] Petkova M.I., Pujol-Carrion N., de la Torre-Ruiz M.A. (2010). Signal flow between CWI/TOR and CWI/RAS in budding yeast under conditions of oxidative stress and glucose starvation. Commun. Integr. Biol..

[45] Sundaram V., Petkova M.I., Pujol-Carrion N., Boada J., de la Torre-Ruiz M.A. (2015). Tor1, Sch9 and PKA downregulation in quiescence rely on Mtl1 to preserve mitochondrial integrity and cell survival. Mol. Microbiol..

[46] Pujol-Carrion N., Pavón-Vergés M., Arroyo J., de la Torre-Ruiz M.A. (2021). The MAPK Slt2/Mpk1 plays a role in iron homeostasis through direct regulation of the transcription factor Aft1. BBA—Mol. Cell Res..

[47] Mitjana F.V., Petkova M.I., Pujol-carrion N., de la Torre-Ruiz M.A. (2011). Pkc1 and actin polymerisation activities play a role in ribosomal gene repression associated with secretion impairment caused by oxidative stress. FEMS Yeast Res..

[48] Petkova M.I., Pujol-Carrion N., de la Torre-Ruiz M.A. (2012). Mtl1 O-mannosylation mediated by both Pmt1 and Pmt2 is important for cell survival under oxidative conditions and TOR blockade. Fungal Genet. Biol..

[49] Niles B.J., Mogri H., Hill A., Vlahakis A., Powers T. (2012). Plasma membrane recruitment and activation of the AGC kinase Ypk1 is mediated by target of rapamycin complex 2 (TORC2) and its effector proteins Slm1 and Slm2. Proc. Natl. Acad. Sci. USA.

[50] Costanzo M., Baryshnikova A., Bellay J., Kim Y., Spear E.D., Sevier C.S., Ding H., Koh J.L.Y., Toufighi K., Mostafavi S. (2010). The Genetic Landscape of a Cell. Science.

[51] Jordá T., Rozès N., Puig S. (2021). Sterol Composition Modulates the Response of *Saccharomyces cerevisiae* to Iron Deficiency. J. Fungi.

[52] Pujol-Carrion N., Gonzalez-Alfonso A., Puig S., Torre-Ruiz M.A., de la Torre-Ruiz M.A. (2021). Both human and soya bean ferritins highly improve the accumulation of bioavailable iron and contribute to extend the chronological life in budding yeast. Microb. Biotechnol..

[53] Kaiser C., Michaelis S., Mitchell A. (1994). Methods in Yeast Genetics.

[54] Mechoud M.A., Pujol-Carrion N., Montella-Manuel S., de la Torre-Ruiz M.A. (2020). Interactions of GMP with Human Glrx3 and with *Saccharomyces cerevisiae* Grx3 and Grx4 Converge in the Regulation of the Gcn2 Pathway. Appl. Environ. Microbiol..

[55] Montella-manuel S., Pujol-carrion N., De Torre-ruiz M.A. (2021). The Cell Wall Integrity Receptor Mtl1 Contributes to Articulate Au-tophagic Responses When Glucose Availability Is Compromised. J. Fungi.

