# Supplementary material for: Aft1 Nuclear Localization and Transcriptional Response to Iron Starvation Rely upon TORC2/Ypk1 Signaling and Sphingolipid Biosynthesis
Source: Int J Mol Sci. 2023 Jan 26;24(3):2438. doi: 10.3390/ijms24032438 (PMC9916926; doi:10.3390/ijms24032438)
Supplement: Supplementary file 1 [file ijms-24-02438-s001.zip › ijms-2178135-supplementary.pdf]

Supplemental 1

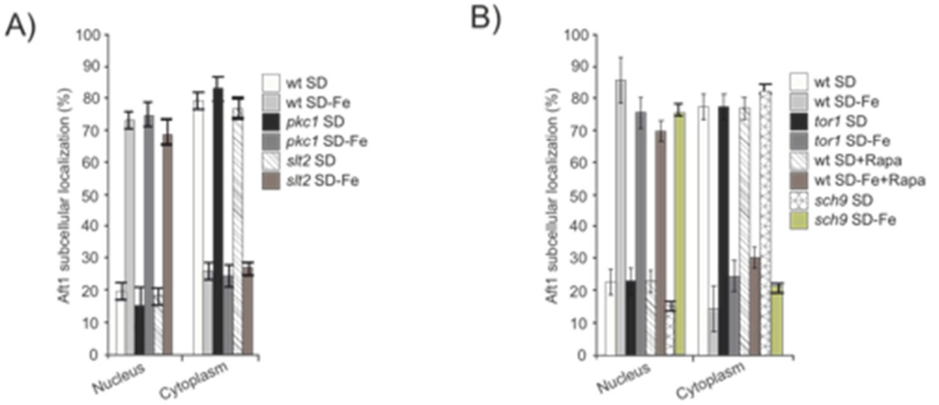

Figure S1. TORC1, Sch9, Pkc1 and Slt2 are not involved in Aft1 nuclear localization. Cultures were grown in SD or SD-Fe to exponential phase at 30ºC. Samples were collected to elucidate Aft1 subcellular localization. **(A)** wt*,* *pkc1* and *slt2* strains transformed with pAft1GFP. **(B)** wt, *tor1* and wt treated with Rapamycin for 2 hours (Rapa) and *sch9* bearing pAft1GFP.

Supplemental 2

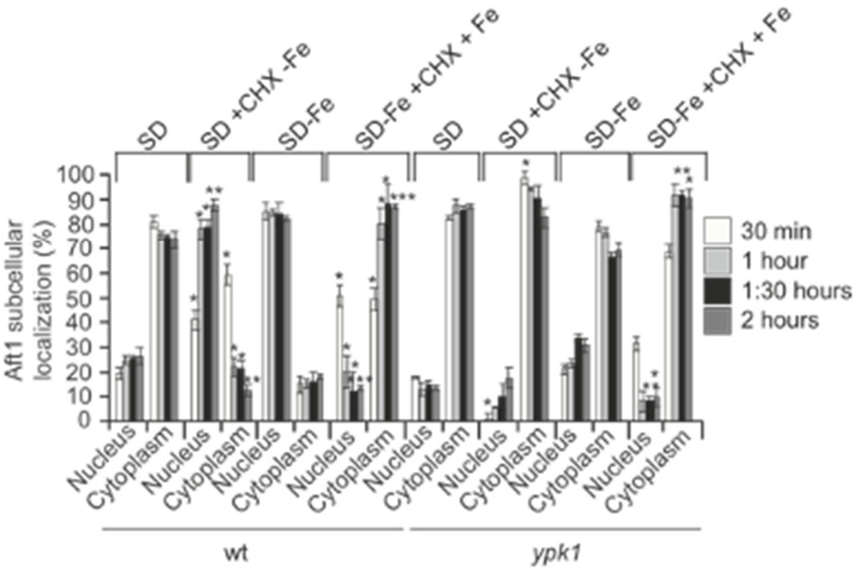

Figure S2. The absence of Ypk1 causes iron regulon deregulation during iron starvation. *In vivo* fluorescence observation at indicated times of wt and *ypk1* strains

bearing pAft1GFP. Cells were grown in SD or SD-Fe media until exponential phase, cultures were divided, cycloheximide (+CHX) was added before to both of them. The SD ones were washed and transferred to a media without iron with CHX; iron (+Fe) and CHX were added to SD-Fe culture.

Supplemental 3

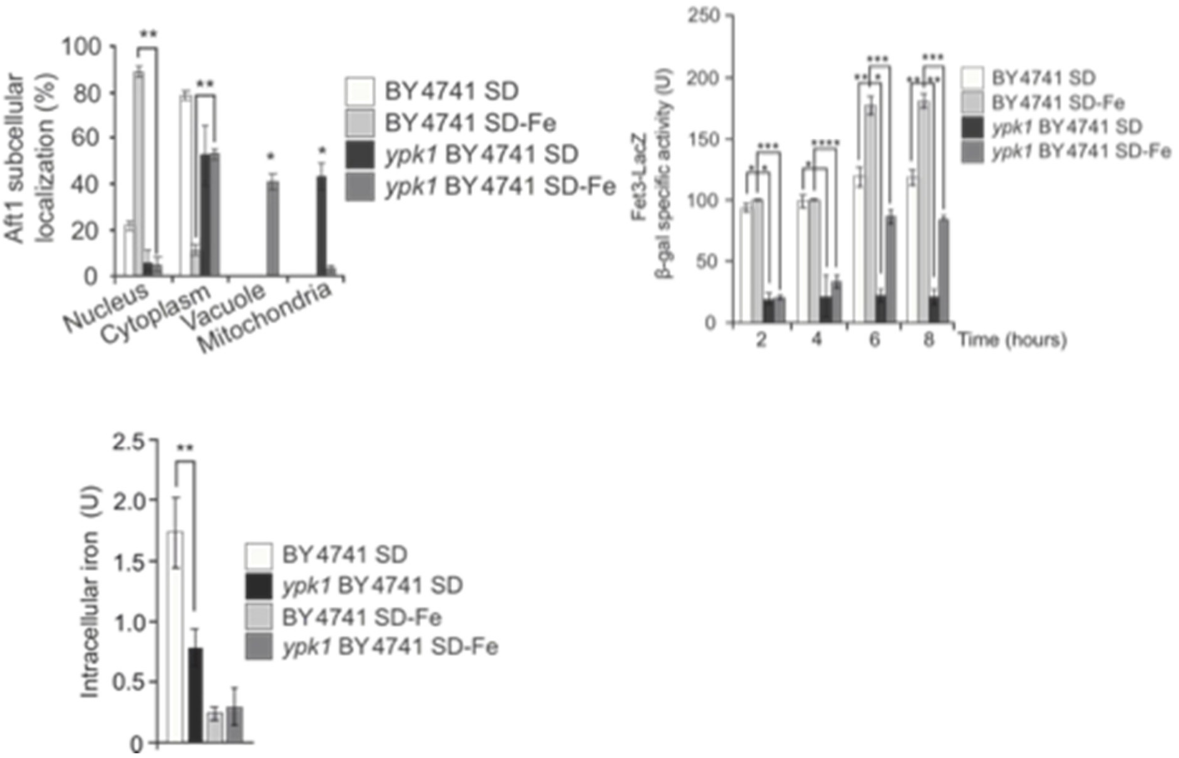

Figure S3. The absence of Ypk1 causes iron regulon deregulation during iron starvation. wt and *ypk1* strains in BY4741 background transformed with the plasmids pAft1GFP and p*FET3*-LacZ. Samples were collected to analyze Aft1 subcellular localization, the ß-galactosidase activity and intracellular iron content. For Aft1 subcellular localization and intracellular iron content cells were logarithmically grown in SD medium plus amino acids or in iron-free SD (SD-Fe). For the ß-galactosidase activity, cells were grown at 30ºC in SD media to OD_{600}:0.4. Aliquots were taken washed and transferred to SD or SD-Fe media for 8 hours. Every 2 hours samples were taken to determine the ß-galactosidase activity reporter construct.

Supplemental 4

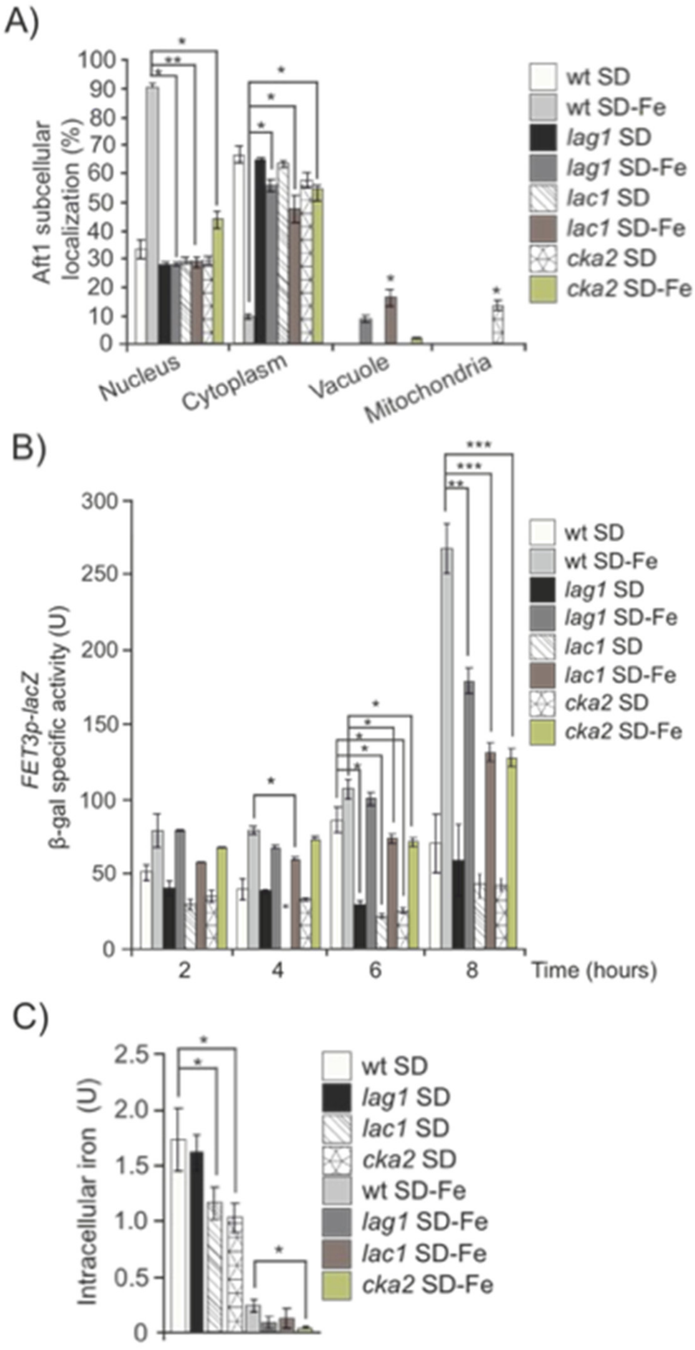

Figure S4. Levels of sphingolipids modify Aft1 localization. **(A)** Aft1 subcellular localization in wt, *lac1, lag1* and *cka2* strains, **(B)** ß-galactosidase activity assay in wt, *lac1, lag1* and *cka2* strains, and **(C)** Intracellular iron assays in wt, *lac1, lag1* and *cka2* strains were performed as in Figure 4.

Supplemental 5

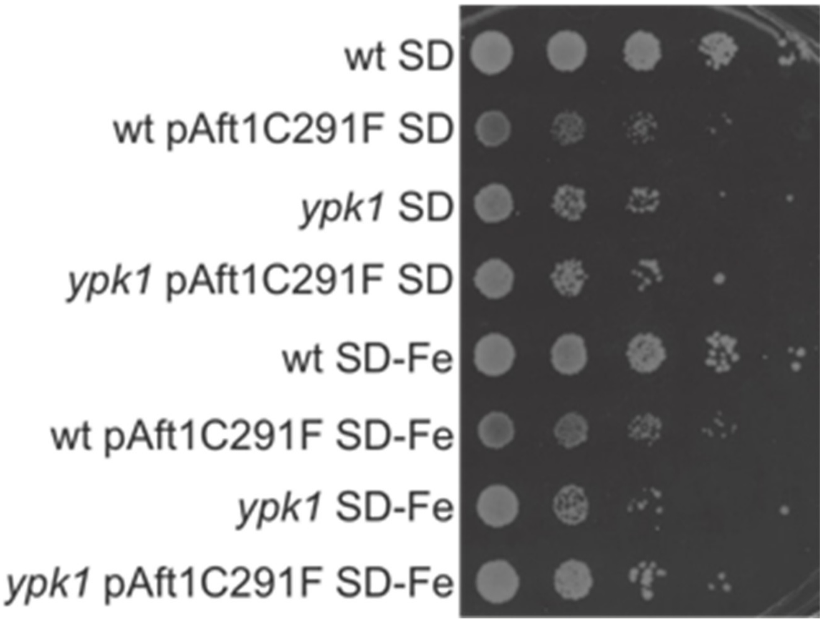

Figure S5. Aft1 nuclear localization is a prosurvival strategy to sphingolipids pathway impairment. wt, wt pAft1C291F, *ypk1* and *ypk1* pAft1C291F cultures were logarithmically grown in SD or SD-Fe medium plus amino acids to be subsequently plated in triplicate on YPD plates. Plates were grown at 30ºC for three days in triplicate, here a representative plate is depicted.
